# Cathepsin D regulates lipid metabolism in murine steatohepatitis

**DOI:** 10.1038/s41598-017-03796-5

**Published:** 2017-06-14

**Authors:** Tom Houben, Yvonne Oligschlaeger, Tim Hendrikx, Albert V. Bitorina, Sofie M. A. Walenbergh, Patrick J. van Gorp, Marion J. J. Gijbels, Silvia Friedrichs, Jogchum Plat, Frank G. Schaap, Dieter Lütjohann, Marten H. Hofker, Ronit Shiri-Sverdlov

**Affiliations:** 1 Departments of Molecular Genetics, Human Biology and Surgery, School of Nutrition and Translational Research in Metabolism (NUTRIM), Maastricht University, 6229 ER Maastricht, The Netherlands; 2 Department of Laboratory Medicine, Medical University of Vienna, 1090 Vienna, Austria; 3 Center for Molecular Medicine (CeMM) of the Austrian Academy of Sciences, 1090 Vienna, Austria; 4 Institute of Clinical Chemistry and Clinical Pharmacology, University of Bonn, D-53127 Bonn, Germany; 5 Molecular Genetics Section, Department of Pediatrics, University Medical Center Groningen, 9700 RB Groningen, The Netherlands

## Abstract

Due to the obesity epidemic, non-alcoholic steatohepatitis (NASH) is a prevalent liver disease, characterized by fat accumulation and inflammation of the liver. However, due to a lack of mechanistic insight, diagnostic and therapeutic options for NASH are poor. Recent evidence has indicated cathepsin D (CTSD), a lysosomal enzyme, as a marker for NASH. Here, we investigated the function of CTSD in NASH by using an *in vivo* and *in vitro* model. In addition to diminished hepatic inflammation, inhibition of CTSD activity dramatically improved lipid metabolism, as demonstrated by decreased plasma and liver levels of both cholesterol and triglycerides. Mechanistically, CTSD inhibition resulted in an increased conversion of cholesterol into bile acids and an elevated excretion of bile acids via the feces, indicating that CTSD influences lipid metabolism. Consistent with these findings, treating *Wt* BMDMs with PepA *in vitro* showed a similar decrease in inflammation and an analogous effect on cholesterol metabolism. ***Conclusion***: CTSD is a key player in the development of hepatic inflammation and dyslipidemia. Therefore, aiming at the inhibition of the activity of CTSD may lead to novel treatments to combat NASH.

## Introduction

Due to the increasing obesity epidemic, non-alcoholic fatty liver disease (NAFLD) has emerged as the most important liver disease in the world^1^. NAFLD comprises a spectrum of liver diseases ranging from benign hepatic steatosis to more advanced liver diseases such as cirrhosis and fibrosis, which eventually might result in liver failure and death. The combination of steatosis and inflammation is referred to as non-alcoholic steatohepatitis (NASH). NASH is a disease stage that can further progress into such advanced liver diseases, underscoring the value of preventing/treating the inflammatory aspect of NAFLD. Currently, the etiology and mechanisms leading to obesity-induced liver inflammation are not clear, resulting in the lack of well-defined effective therapies^2, 3^.

Using mice lacking the low-density lipoprotein receptor (*Ldlr* ^−/−^), we previously demonstrated a positive correlation between hepatic inflammation and cholesterol accumulation in lysosomes of Kupffer cells (KCs)^4, 5^. Moreover, lysosomal enzymes such as cathepsin D (CTSD) are increasingly recognized for their involvement in inflammatory responses^6, 7^. Whereas it was initially believed that proteases such as CTSD were only involved in non-specific protein degradation inside acidic lysosomes, research of the past decade has indicated that CTSD has a wide spectrum of functions, both of physiological and pathological nature^8, 9^. Under pathological inflammatory conditions, CTSD has been associated with distinct disorders such as rheumatoid arthritis, Alzheimer’s disease, atherosclerosis and inflammatory bowel disease (IBD)^10–13^. Furthermore, recent data also revealed a correlation between hepatic inflammation and the activity and expression of CTSD in the liver^14–16^. Relevantly, plasma CTSD levels correlated with different stages of NAFLD^17^. These data suggest the involvement of lysosomes, and more specifically the lysosomal enzyme CTSD, in mediating the inflammatory response in NASH. However, despite the established relationship between lysosomes and inflammation as well as the awareness of an association between the lysosomal enzyme CTSD and hepatic inflammation, the exact function of CTSD in the context of NASH has not yet been investigated.

In the current study, we hypothesized that proteolytic inhibition of CTSD leads to reduced steatohepatitis. To induce NASH, *Ldlr* ^−/−^ mice were put on a high-fat, high-cholesterol (HFC) diet for three weeks. This model was chosen due to its close resemblance with a human-like lipoprotein profile combined with the presence of hepatic inflammation, thereby serving as a physiological model to study NASH^18^. To examine whether inhibition of CTSD decreases hepatic inflammation, *Ldlr* ^−/−^ mice were injected with pepstatin A (PepA), an inhibitor of aspartyl proteases, for three weeks or only in the final week of the experiment. In agreement with our hypothesis, inhibition of the proteolytic activity of CTSD reduced hepatic inflammation. Remarkably, proteolytic inhibition of CTSD dramatically improved lipid and lipoprotein metabolism, which is demonstrated by decreased plasma and liver levels of both cholesterol and triglycerides. Mechanistically, CTSD inhibition resulted in an increased conversion of cholesterol into bile acids as well as an elevated excretion of bile acids via the feces, indicating that CTSD influences lipid metabolism. Consistent with these findings, treating wildtype (*Wt*) bone marrow-derived macrophages (BMDMs) with PepA showed a similar decrease in inflammation and an analogous effect on cholesterol metabolism. These data demonstrate for the first time a key regulatory role for CTSD in lipid metabolism in the development of NASH. Hence, aiming at the modulation of CTSD activity in NASH holds therapeutic promise and should be further investigated in the future.

## Results

### Decreased systemic and hepatic inflammation in Ldlr^−/−^ mice after inhibition of CTSD

**Figure 1:**
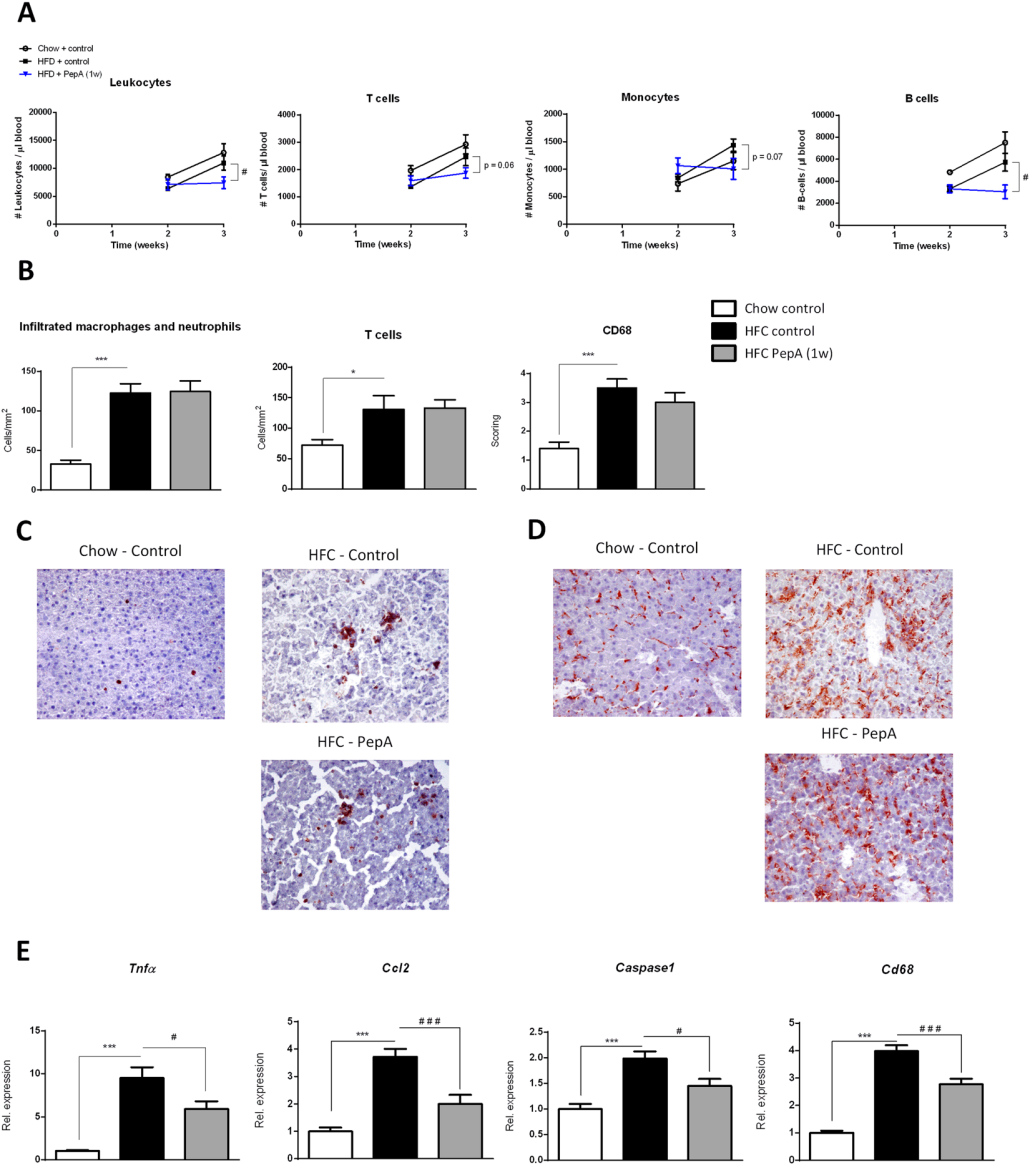
Parameters of systemic and hepatic inflammation in control- and PepA-injected mice. (**A**) Absolute levels of leukocyte, T cell, monocyte and B cell populations at week 2 and 3 of the experiment. (**B**) Liver sections were stained for infiltrating macrophages and neutrophils (Mac-1), T cells (CD3) and resident monocytes/macrophages (CD68). Positive cells were counted (infiltrated macrophages/neutrophils and T cells) or scored (CD68). (**C,D**) Representative pictures of the Mac-1 (**C**) and CD68 staining (**D**) (original magnification, 200x). (**E**) Gene expression analysis of *Tnfα*, *Ccl2*, *Caspase1* and *Cd68*. Data are shown relative to control mice on chow diet. Error bars represent ± SEM; *indicates *p* ≤ 0.05 and ***p < 0.001 compared to mice on chow diet; ^#^indicates *p* ≤ 0.05 and ^###^ *p* < 0.001 compared to control-injected mice on HFC diet by use of two-tailed unpaired *t* test; for FACS n = 4 animals per group and for immunohistochemistry and gene expression n = 8–11 animals per group.

To elucidate the systemic immune effects of proteolytic inhibition of CTSD, blood leukocyte levels were determined via fluorescence-activated cell sorting after two weeks and at the end of the experiment. Administration of PepA for one week to *Ldlr* ^−/−^ mice on HFC diet resulted in a significant decrease of blood leukocyte, B and CD4^+^ T cells after 3 weeks (Fig. 1A and Supplementary Fig. S2). Though not significant, similar trends were also observed for T cells, monocytes, NK cells, Ly6C^high^ monocytes, Ly6c^int^ monocytes, Ly6C^low^ monocytes and CD8^+^ T cells, confirming the reduced systemic inflammatory phenotype after proteolytic inhibition of CTSD (Fig. 1A and Supplementary Fig. S2). In contrast, no statistical differences were observed in blood granulocyte levels between the groups (Supplementary Fig. S2).

To determine the effect of the proteolytic inhibition of CTSD on hepatic inflammation, immunohistochemical stainings were performed on liver sections for the inflammatory cell markers Mac-1, CD3, CD68 and F4/80. Quantification of all immunohistochemical stainings showed no effect of one week PepA treatment on hepatic inflammation in *Ldlr* ^−/−^ mice on HFC diet at histological level (Fig. 1B–D and Supplementary Table S1). This observation was also confirmed by H&E-staining (see Supplementary Fig. S3A,B). Additionally, plasma alanine transaminase levels, indicative for liver damage, did not change upon one week PepA treatment (data not shown).

As inflammatory changes at histological level usually require administration of the therapeutic agent for more than one week before these effects can be observed^19–21^, we also performed hepatic gene expression analysis on the inflammatory markers tumor necrosis factor alpha (*Tnfα*), chemokine (C-X-C motif) ligand-2 *(Ccl2)*, *Caspase1*, cluster of differentiation 68 (*Cd68*), interleukin-12 (*Il12*) and vascular cell adhesion protein (*Vcam*). As shown in Fig. 1E, Supplementary Fig. S3D and Supplementary Table S1, when *Ldlr* ^−/−^ mice on HFC diet were treated with PepA for one week, hepatic gene expression levels of *Tnfα*, *Ccl2*, *Caspase1*, *Cd68* and *VCAM* were significantly reduced compared to control-treated *Ldlr* ^−/−^ mice on HFC diet (Fig. 1E). A similar trend was also observed for hepatic gene expression of *IL12* and for the M2 markers cluster of differentiation 206 (*Cd206*), Early growth response protein 2 (*Egr2*) and ratio of inducible nitric oxide (*iNos*) and arginase 1 (*Arg1*) (see Supplementary Fig. 3C and Supplementary Table S1). Accordingly, hepatic protein levels of the inflammatory cytokines TNFα and IL12 were also reduced upon PepA administration (see Supplementary Table S1), indicating CTSD as a contributing compound in evoking the hepatic inflammatory response. Moreover, when injected over the total three weeks, *Ldlr* ^−/−^ mice showed a nearly significant decrease of hepatic T cell levels, which was combined with an even stronger reduction of hepatic inflammatory markers at gene and protein expression level (see Supplementary Table S1). Altogether, these data demonstrate that inhibition of the proteolytic site of CTSD in *Ldlr* ^−/−^ mice reduces the development of systemic inflammation and hepatic inflammatory gene expression, thereby pointing to a pro-inflammatory role of CTSD.

### Improved hepatic lysosomal function after inhibition of intracellular and circulating CTSD is linked to hepatic inflammation in Ldlr^−/−^ mice

**Figure 2:**
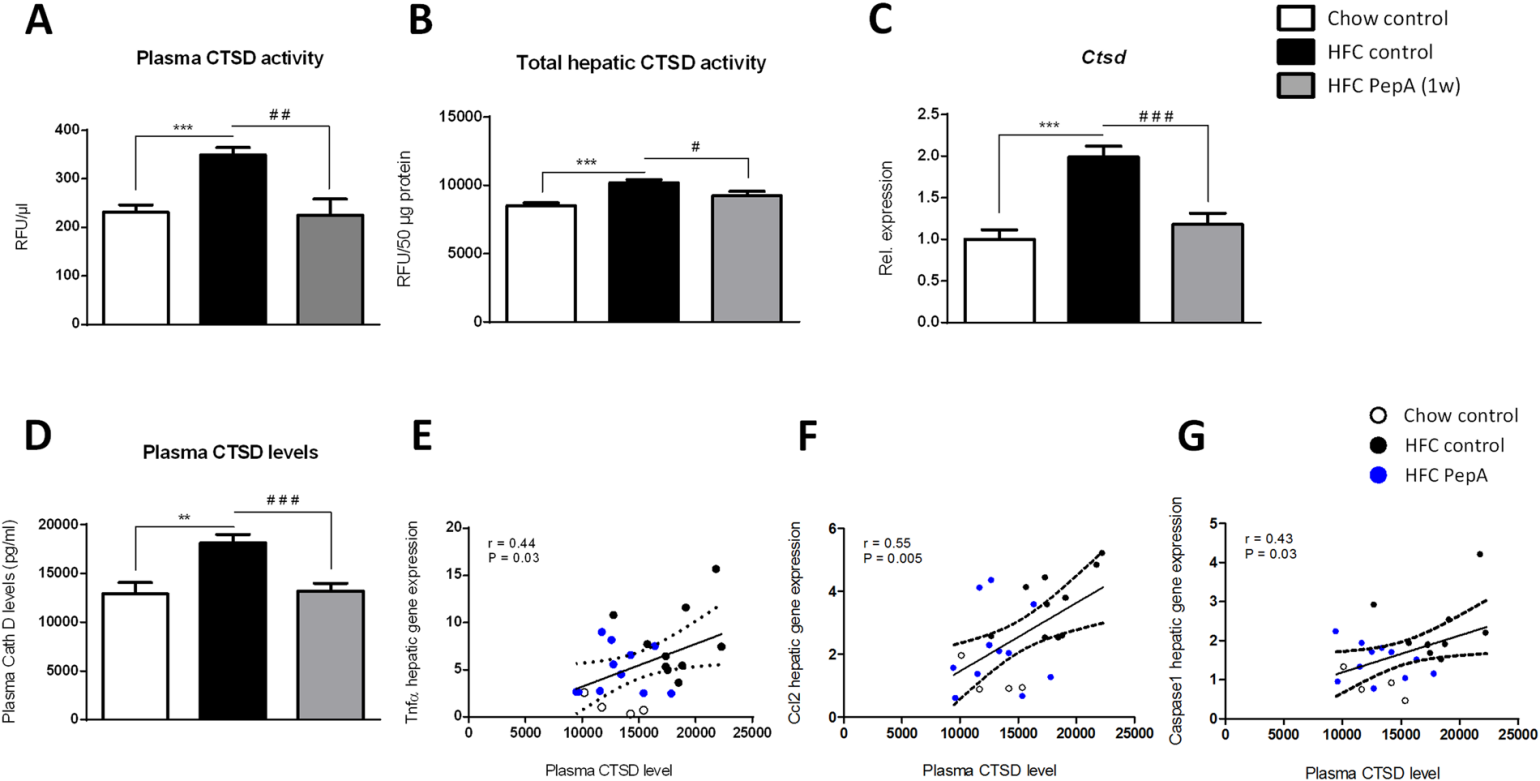
Parameters of lysosomal function in control- and PepA-injected mice. (**A,B**) Plasma (**A**) and hepatic (**B**) activity of CTSD. (**C**) Hepatic gene expression analysis of *Ctsd*. Data are shown relative to control mice on chow diet by use of two-tailed unpaired *t* test. (**D**) Plasma levels of CTSD. (**E–G**) Correlation between plasma levels of CTSD and hepatic gene expression of *Tnfα*, *Ccl2* and *Caspase1* by Pearson correlation. Error bars represent ± SEM. **Indicates *p* < 0.01 and ***p < 0.001 compared to mice on chow diet; ^#^indicates *p* ≤ 0.05, ^##^ *p* < 0.01 and ^###^ *p* < 0.001 compared to control-injected mice on HFC diet by use of two-tailed unpaired *t* test. n = 9–11 animals in each group. For correlation analysis, n = 4 animals on chow diet and n = 10 for mice on HFC diet.

To investigate the effect of PepA on hepatic lysosomal function in diet-induced hepatic inflammation, plasma and hepatic CTSD activity as well as the activity the lysosomal enzyme acid phosphatase (AP) were measured in total liver. Treating *Ldlr* ^−/−^ mice on HFC diet with PepA for one week resulted in a striking decrease of plasma CTSD activity compared to control-treated mice (Fig. 2A). Furthermore, PepA-treated *Ldlr* ^−/−^ mice on HFC diet demonstrated a reduction in hepatic CTSD activity (Fig. 2B). In contrast, one week PepA-treatment did neither affect plasma nor hepatic cathepsin E (CTSE) activity (Supplementary Fig. 4A,B). Collectively, these results indicate that PepA is a proteolytic inhibitor of intracellular and circulating CTSD. Of note, while *Ldlr* ^−/−^ mice on a HFC diet showed increased plasma and hepatic CTSD activity compared to *Ldlr* ^−/−^ mice on chow diet (Fig. 2A,B), when compared to *Wt* mice on HFC diet, plasma CTSD (and not hepatic CTSD) activity increased in HFC-fed *Ldlr* ^−/−^ mice (data not shown). These data suggest that circulating CTSD likely plays the most important role in developing hepatic inflammation.

Subsequently, the activity of acid phosphatase (AP) was measured in the liver. Although total hepatic AP activity was similar among all groups, control-treated mice on a HFC diet for 3 weeks showed reduced levels of lysosomal AP activity compared to mice on chow diet, confirming lysosomal dysfunction upon HFC diet (see Supplementary Fig. S4C,D). In contrast, PepA-treated *Ldlr* ^−/−^ mice on HFC diet demonstrated elevated levels of hepatic lysosomal AP activity compared to control mice on HFC diet (see Supplementary Fig. S4D). To confirm these effects on lysosomal function, hepatic gene expression was measured for the lysosomal enzymes cathepsin D (*Ctsd*), cathepsin S (*Ctss*) and *Ap*. As shown in Fig. 2C and Supplementary Fig. S4E, hepatic gene expression of *Ctsd*, *Ctss* and *Ap* returned to chow levels after inhibition of CTSD, confirming the rebalancing of lysosomal function after CTSD inhibition. Additionally, to validate whether plasma CTSD levels correlate with the level of hepatic inflammation, plasma CTSD levels were measured. *Ldlr* ^−/−^ mice on an HFC diet showed increased levels of plasma CTSD levels, whereas mice that received the PepA treatment, showed reduced plasma CTSD levels (Fig. 2D). Moreover, hepatic gene expression levels of the inflammatory markers *Tnfα*, *Ccl2* and *Caspase1* correlated significantly with plasma levels of CTSD (Fig. 2E), underlining the link between hepatic inflammation and plasma CTSD levels. Together, these data reveal that proteolytic inhibition of intracellular and circulating CTSD improves diet-induced hepatic lysosomal dysfunction, which is connected to hepatic inflammation in *Ldlr* ^−/−^ mice.

### Improved lipid and lipoprotein metabolism in PepA-treated Ldlr^−/−^ mice on HFC diet

**Figure 3:**
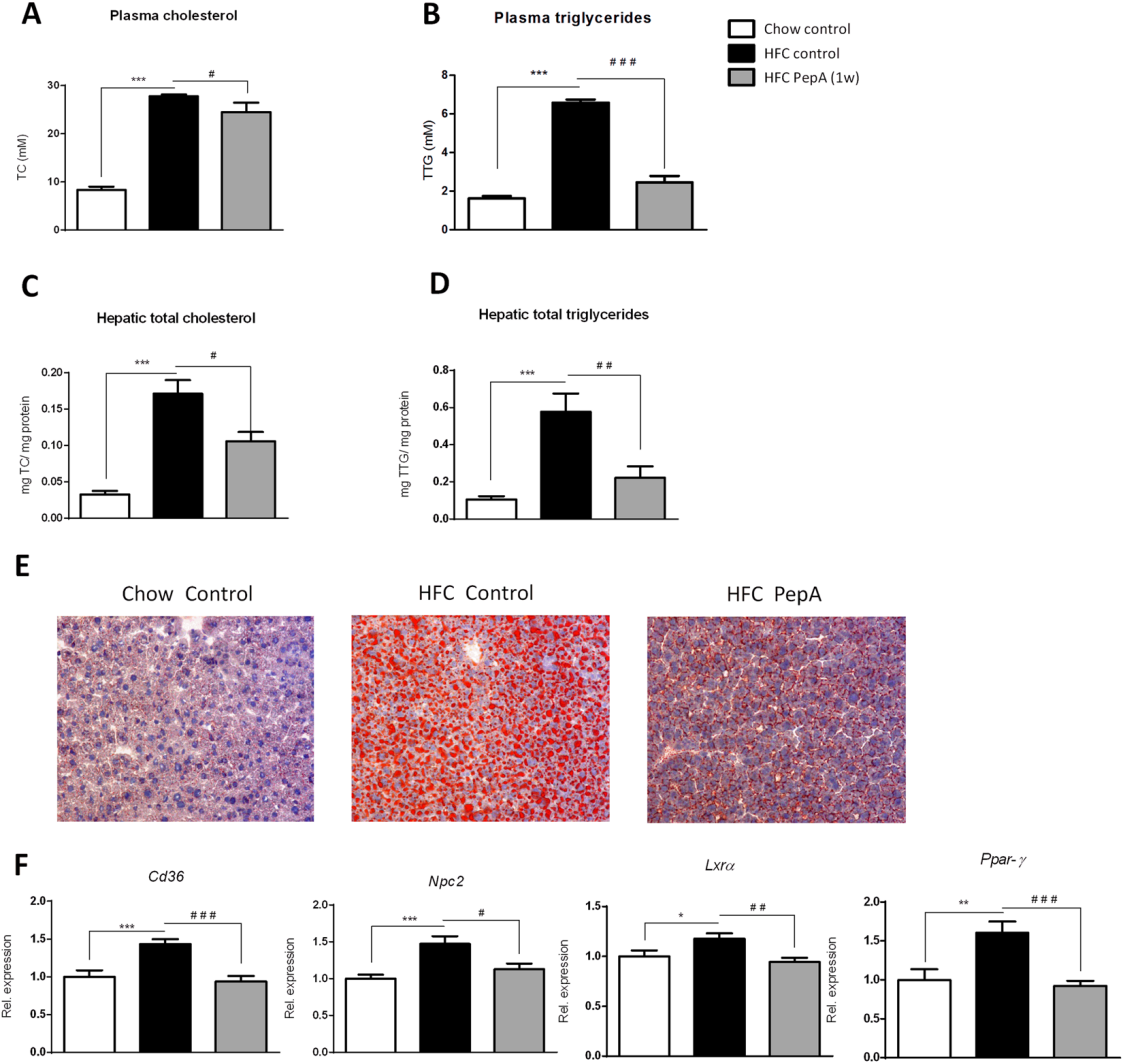
Plasma and hepatic lipids in hyperlipidemic mice with or without PepA-treatment. (**A,B**) Plasma total cholesterol and triglyceride measurements. (**C,D**) Total hepatic cholesterol and hepatic triglyceride measurements. (**E**) Representative pictures (original magnification, 200x) of the Oil red O staining. (**F**) Gene expression analysis of *Cd36*, *Npc2*, *Lxrα* and *Ppar-γ*. Gene expression data are shown relative to control mice on chow diet. Error bars represent ± SEM; *indicates *p* ≤ 0.05, **p < 0.01 and ***p < 0.001 compared to mice on chow diet; ^#^indicates *p* ≤ 0.05, ^##^ *p* < 0.01 and ^###^ *p* < 0.001 compared to control-injected mice on HFC diet by use of two-tailed unpaired *t* test. n = 9–11 animals for each group analysis.

To determine whether inhibition of CTSD affects lipid metabolism, plasma and hepatic lipid levels were measured. Whereas plasma cholesterol and triglycerides were elevated upon feeding an HFC diet, administration of PepA significantly decreased both cholesterol and triglycerides in the plasma (Fig. 3A,B). As shown in the fast protein liquid chromatography (FPLC) profiles, the reductions of both lipids were mainly attributed to a decrease in the VLDL fraction (see Supplementary Fig. S5A–D). Similar to plasma cholesterol and triglyceride levels, hepatic cholesterol and triglyceride levels were also elevated in control-treated *Ldlr* ^−/−^ mice on a HFC diet and decreased upon treatment with PepA (Fig. 3C,D). These changes in hepatic lipid levels were confirmed by Oil red O staining (Fig. 3E and Supplementary Fig. S5E) and are in line with the changes observed in the relative liver weights of these mice (Supplementary Fig. S5F).

To further define the influences of PepA-mediated CTSD inhibition on hepatic lipid metabolism, hepatic gene expression analysis was performed on markers involved in lipid homeostasis. Compared to control-injected *Ldlr* ^−/−^ mice on HFC diet, PepA administration resulted in reduced gene expression levels of Cluster of differentiation 36 (*Cd36*), Niemann-Pick C2 (*Npc2*) and Liver X receptor alpha (*Lxrα*), suggesting a reduced uptake, intralysosomal presence and efflux of cholesterol in these mice respectively (Fig. 3F). In addition, *peroxisome proliferator-activated receptor gamma (Ppar-γ)* expression levels, a lipid-activated transcription factor of genes controlling lipid metabolism, were also decreased upon PepA administration, indicating a dramatic improvement in lipid homeostasis (Fig. 3F).

**Figure 4:**
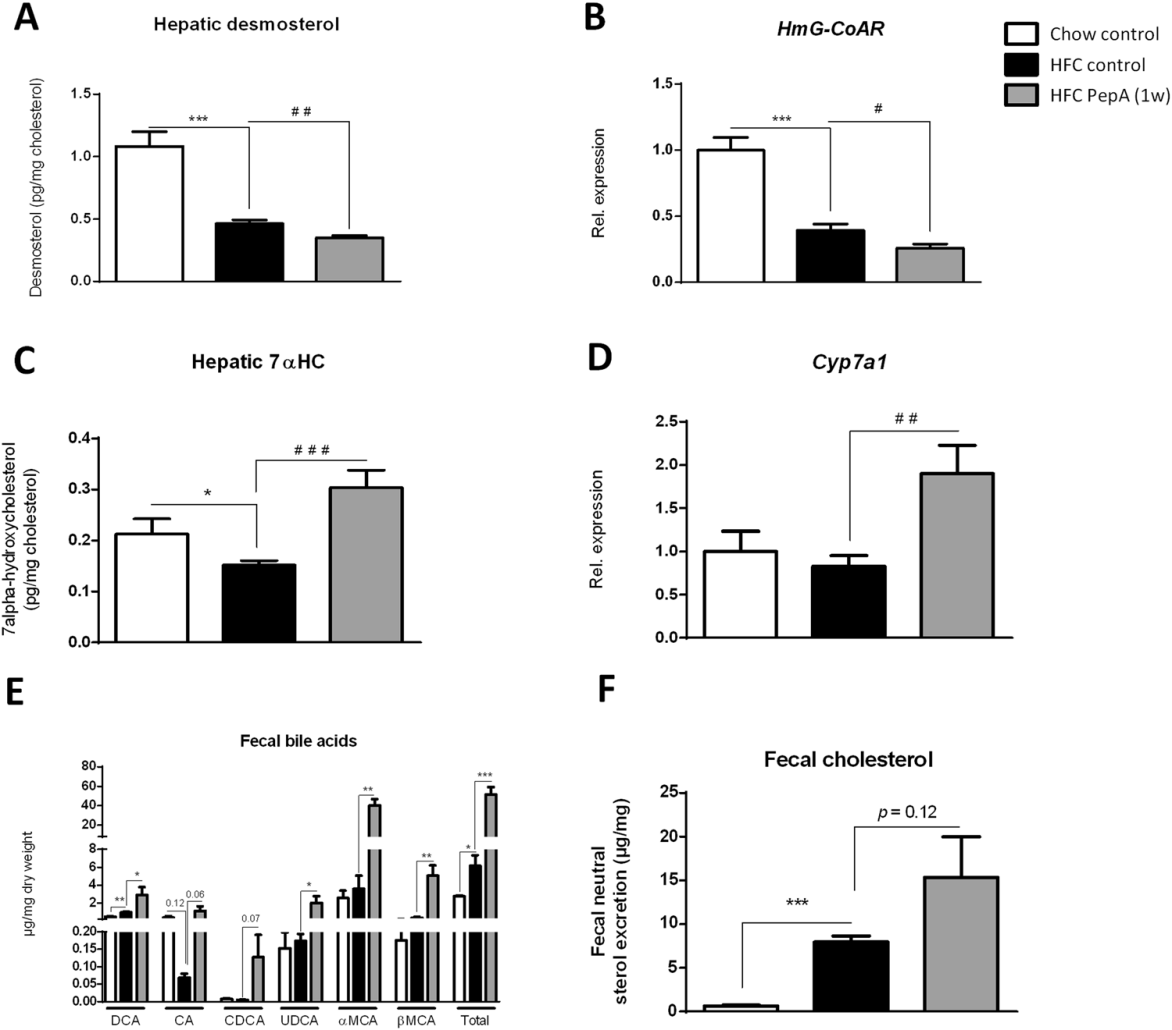
Hepatic *de novo* synthesis and degradation of cholesterol. (**A,C**) Hepatic concentrations of desmosterol (**A**) and 7 alpha-hydroxycholesterol (7αHC; **C**). Both values are shown relative to the hepatic cholesterol concentrations. (**B,D**) Gene expression analysis of *HmG-CoAR* and *Cyp7a1*. (**E,F**) Fecal bile acid (**E**) and cholesterol levels (**F**). Both measurements are relative to mg dry weight of the fecal sample. Gene expression data are shown relative to control mice on chow diet. Error bars represent ± SEM; *indicates *p* ≤ 0.05 and ***p < 0.001 compared to mice on chow diet; ^#^indicates *p* ≤ 0.05, ^##^ *p* < 0.01 and ^###^ *p* < 0.001 compared to control-injected mice on HFC diet by use of two-tailed unpaired *t* test; n = 11 for each group. DCA, deoxycholic acid; CA, cholic acid; CDCA, chenodeoxycholic acid; UDCA, ursodeoxycholic acid; αMCA, α-muricholic acid; βMCA, β-muricholic acid.

As the proteolytic inhibition of CTSD also appeared to influence hepatic cholesterol metabolism, hepatic synthesis and degradation of cholesterol were investigated in more detail. Inhibition of CTSD by PepA decreased hepatic cholesterol synthesis, as shown by measurements of hepatic desmosterol levels (Fig. 4A). This finding was confirmed by gene expression levels of the rate-limiting enzyme for cholesterol synthesis, *3-hydroxy-3-methyl-glutaryl-Coenzyme A reductase* (*HmG-CoAR*) (Fig. 4B). Besides cholesterol synthesis, also cholesterol degradation was affected upon inhibition of CTSD. Specifically, whereas also 27-hydroxcholesterol (27HC; see Supplementary Fig. S6A) levels were nearly significantly elevated, the conversion of cholesterol into 7 alpha-hydroxycholesterol (7αHC) was significantly increased in PepA-injected *Ldlr* ^−/−^ mice on a HFC diet compared to control-injected mice on HFD diet (Fig. 4C). In line, hepatic gene expression levels of the cytochrome P450 7A1 (*Cyp7a1*) enzyme, responsible for this conversion, were elevated in PepA-treated *Ldlr* ^−/−^ mice on a HFC diet, corroborating increased cholesterol degradation after inhibition of CTSD (Fig. 4D). Furthermore, whereas plasma and hepatic bile acid levels remained unaffected (see Supplementary Fig. S6B and C), PepA-treatment increased fecal bile acid (significant) (Fig. 4E) and cholesterol (not significant) levels (Fig. 4F). Overall, these data indicate that inhibition of CTSD activity results in increased bile acid synthesis eventually leading to excretion via the feces, thereby indicating an effect of CTSD on lipid metabolism.

### Inhibition of the proteolytic function of CTSD reduces inflammation in oxLDL-loaded BMDMs

**Figure 5:**
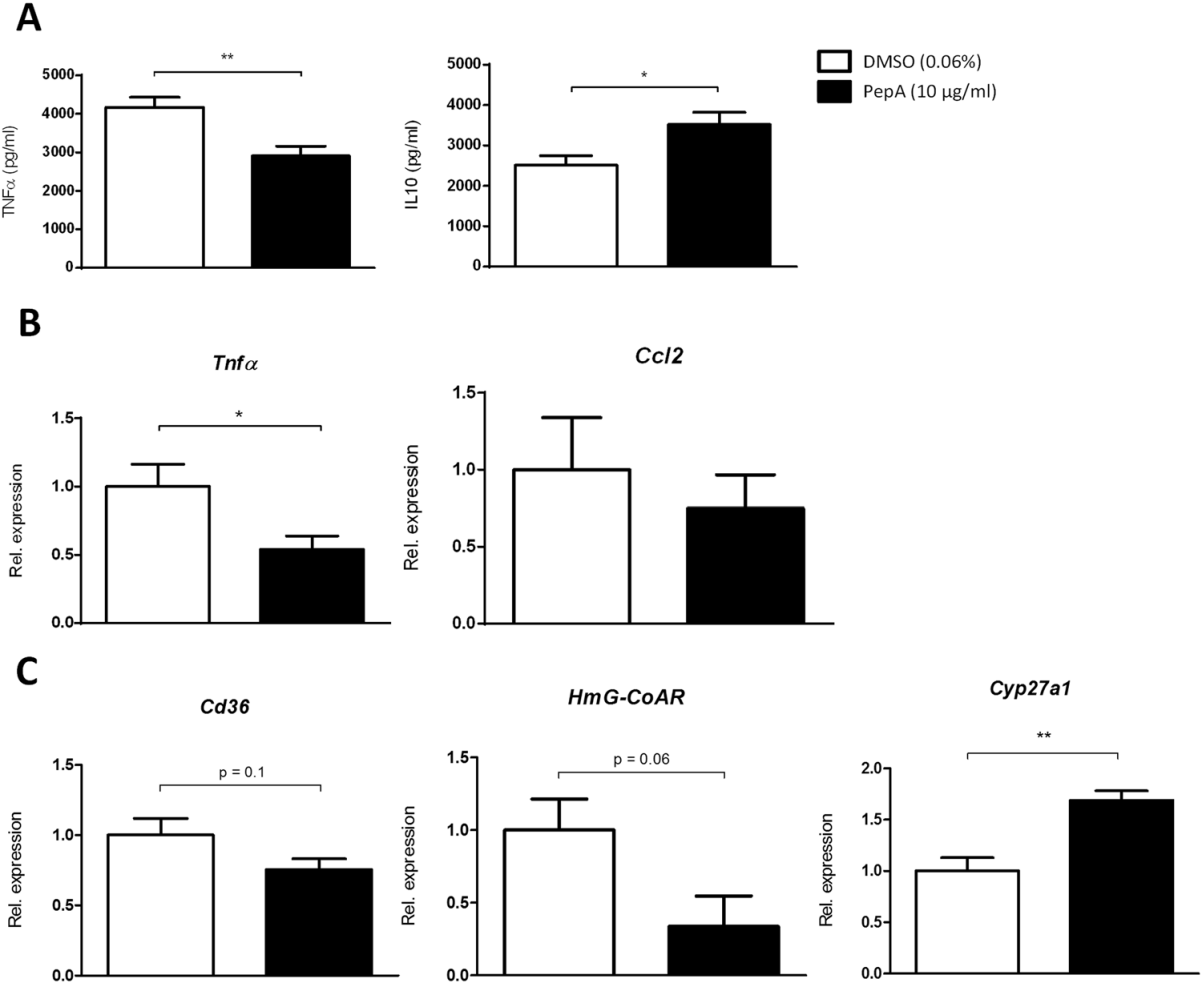
Gene expression and cytokine levels of oxLDL-loaded bone marrow-derived macrophages (BMDM) treated with PepA. (**A**) TNFα and IL10 cytokine secretion in supernatant of *Wt* mouse BMDMs, exposed to oxLDL for 24 hr and, subsequently, treated with or without PepA (4 hr) and LPS (4 hr). (**B,C**) Gene expression analysis of *Tnfα*, *Ccl2, Cd36, HmG-CoAR* and *Cyp27a1* measured in *Wt* mouse BMDMs. Gene expression data are shown relative to DMSO-treated BMDMs. All data represent the mean value of three or four independent experiments (with n = 13-16 per group). Error bars represent ± SEM; *indicates *p* ≤ 0.05, **p < 0.01 and ***p < 0.001 compared to DMSO-treated bone marrow-derived macrophages by use of two-tailed unpaired *t* test.

In order to explore the specific role of macrophages on the reduced inflammatory response upon CTSD inhibition, bone marrow cells of *Wt* mice were isolated, differentiated to macrophages and incubated with oxLDL for 24 hr. Subsequently, cells were treated with PepA for 4 hr, followed by 4 hr stimulation with lipopolysaccharide (LPS). Upon incubation with PepA, cytokine levels of the pro-inflammatory marker TNFα, measured in the supernatant of the BMDMs, were significantly reduced compared to carrier (dimethyl sulfoxide (DMSO))-treated cells (Fig. 5A, *left panel*). Additionally, cytokine levels of the anti-inflammatory marker IL10 increased after PepA treatment (Fig. 5A, *right panel*), confirming the reduction in inflammation upon PepA treatment and the pro-inflammatory properties of CTSD in lipid-induced inflammation (as CTSE activity was also here not affected by PepA (see Supplementary Fig. S7). In line, *Tnfα* gene expression measured in BMDMs showed a decrease after PepA treatment. A similar trend was also observed for the expression of *Ccl2*, but this did not reach statistical significance (Fig. 5B, *right panel*). The aggregated *in vitro* findings are in line with a reduction in hepatic inflammation upon PepA treatment, and by extension a pro-inflammatory action of CTSD.

To study whether the PepA-dependent reduction in inflammation was accompanied by an improvement in cholesterol metabolism, gene expression analysis was performed for *Cd36*, *Cyp27a1* and *HmG-CoAR*. In line with the hepatic *in vivo* gene expression data, cholesterol uptake (*Cd36*) and synthesis (*HmG-CoAR*) were reduced, though not significant, after PepA treatment in BMDMs. Next, *Cyp27a1*, the main enzyme responsible for the degradation of cholesterol in macrophages, appeared to be upregulated, indicating higher levels of cholesterol degradation in BMDMs (Fig. 5C). Thus, oxLDL-loaded BMDMs demonstrate a reduced inflammatory response upon proteolytic inhibition of CTSD, likely caused by an improved cholesterol metabolism.

## Discussion

A major impediment to the development of well-defined, effective therapies for NASH has been the lack of understanding the mechanisms leading to the inflammatory component of this disorder. In the current study, we show for the first time that the lysosomal enzyme CTSD is a key player in the pathogenesis of NASH. Mechanistically, our data suggest that the pro-inflammatory function of CTSD is tied to a regulation of general lipid metabolism. In addition, we provide evidence for the central role of macrophages in exerting the lipid-induced effects of CTSD in the liver. These findings unravel CTSD as a promising, novel target for the treatment of NASH.

While CTSD was shown to have anti-inflammatory properties in the context of neutrophils^22^, ample evidence points towards the pro-inflammatory effects of CTSD. In human intestinal epithelial cells and a mouse model for IBD, it was shown that activation of the lysosomal enzyme CTSD contributes to intestinal inflammation^12, 23, 24^. Furthermore, inhibition of CTSD and CTSS reduced cardiovascular inflammation and attenuated atherosclerotic lesion progression, reiterating the contribution of cathepsins to pathological inflammatory responses^7, 11, 25, 26^. Notably, in the aforementioned IBD and atherosclerotic studies, the pro-inflammatory effects of CTSD were suggested to be derived from macrophages^7, 12, 23, 24^. Indeed, lysosomal enzymes in macrophages appear to play a critical role in withstanding the lipid challenge in obesity-induced inflammatory processes^27, 28^. In line, cytokine release was reduced in oxLDL-loaded BMDMs treated with PepA, substantiating the contribution of macrophages to CTSD-mediated hepatic inflammation. Surprisingly, hepatic macrophage levels (indicated by CD68 quantification) were not reduced after CTSD inhibition. Potential explanations for this finding might be a delayed egress of macrophages from the liver upon resolution of inflammation^21^ or due to a prolonged half-life of macrophages^20, 29^. Relevantly, while we demonstrate a positive correlation between hepatic CTSD expression and hepatic inflammation, Fukuo *et al*. showed reduced levels of hepatic CTSD in NAFLD patients^14^. However, these patients were not evaluated for the presence of inflammation and, thereby, cannot be used to link hepatic inflammation to reduced CTSD expression. Together, our current observations are in line with a causal relation between lipid-induced lysosomal dysfunction in hepatic macrophages and the level of inflammation in the liver^15^. Thus, our data point towards a pivotal role for macrophage-derived CTSD in the development of hepatic inflammation.

Relevantly, proteolytic inhibition of CTSD resulted in reduced cholesterol and triglyceride levels in plasma and liver. This finding suggests that the improved NASH-phenotype in PepA-treated *Ldlr* ^−/−^ mice is due to an effect of CTSD on lipid metabolism. In agreement with this finding, prevention of the proteolytic activation of the lysosomal enzyme acid sphingomyelinase was shown to reduce cholesterol and triglyceride levels in the liver^30^. Therefore, the influence of CTSD on lipid metabolism, as observed in this study, might not be unique to CTSD, but could rather be a shared mechanism of several lysosomal enzymes to control lipid homeostasis. In support of this concept, a deficiency in the gene encoding for CTSD results in a specific type of Batten disease, a disorder which is characterized by the cellular accumulation of lipid-containing residues^31, 32^.

**Figure 6:**
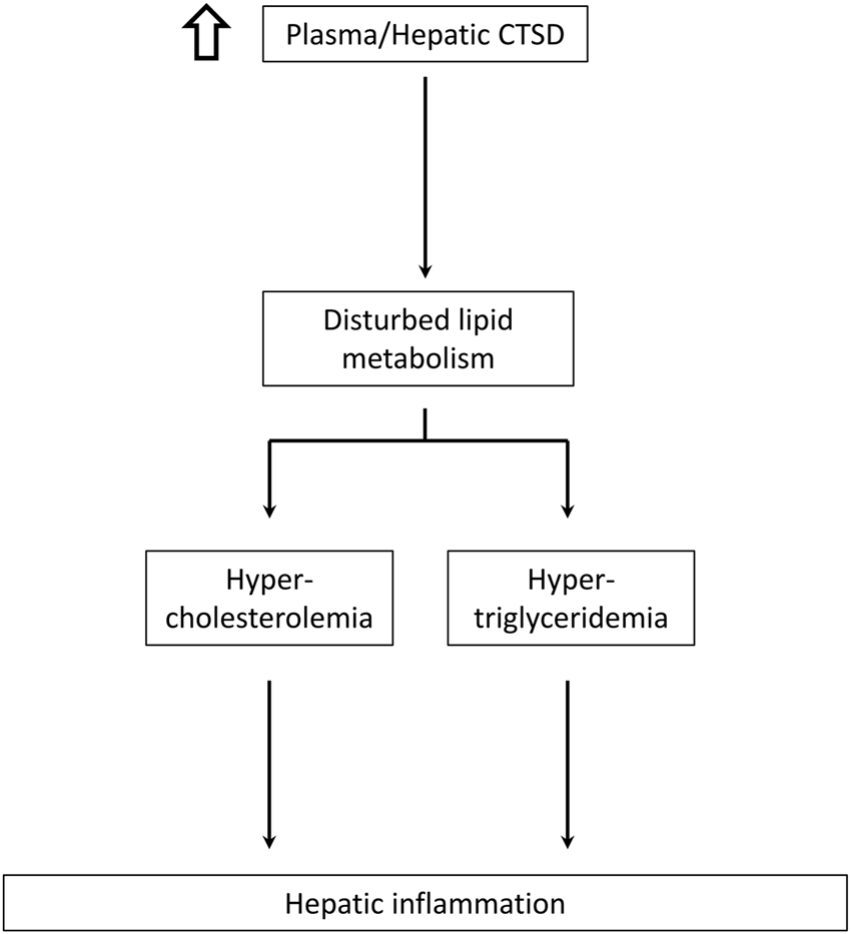
Schematic overview illustrating the proposed by which CTSD participates in the development of hepatic inflammation. Based on findings of the current study, increased levels of CTSD lead to a disturbance in lipid metabolism, which is known to result in increased plasma cholesterol (hypercholesterolemia) and triglyceride (hypertriglyceridemia) levels. Consequently, hypercholesterolemia and hypertriglyceridemia can lead to hepatic inflammation.

There is considerable evidence contributing to the concept that a disturbance in bile acid metabolism interferes with cholesterol and, by extension, triglyceride metabolism. For example, a deficiency in the enzyme CYP7A1 was shown to lead to a hypercholesterolemic phenotype^33^, which is in line with our results that showed the same inverse relation between *Cyp7a1* and plasma cholesterol levels. Of note, patients with CYP7A1 deficiency were also reported to be hypertriglyceridemic. In agreement, triglyceride homeostasis and bile acid metabolism were revealed to be closely intertwined by regulation of farnesoid X receptor (FXR) and small heterodimer partner (SHP) on sterol regulatory element-binding protein (SREBP-1c), which is a known transcription factor of fatty acid and cholesterol synthesis^34, 35^. Similar to cholesterol levels, plasma and liver triglyceride levels in our study were inversely correlated with 7αHC levels, the first precursor of the classical pathway of bile acid formation. In addition, hypertriglyceridemia has been associated with increased systemic inflammation^36^. Therefore, the overall observed effects on lipid metabolism in our study might be explained by a direct link between CTSD and bile acid metabolism. In agreement with this concept, though bile acids levels in plasma and liver were similar, fecal levels were dramatically increased after CTSD inhibition. Therefore, our data indicate that modulation of CTSD has the potential to increase bile acid excretion, resulting in reduced lipid levels in hyperlipidemic conditions (Fig. 6).

The observation that both plasma (circulating) and hepatic (intracellular) CTSD activity were decreased after PepA inhibition suggests that the results in this study are derived from intra- and extracellular effects of CTSD. Indeed, intracellular CTSD-induced apoptosis was demonstrated to induce inflammation in the liver^37^. Therefore, though the strongest decrease in activity was observed in the circulating fraction of CTSD, the observed effects are likely a combination of the modulation of intra- and extracellular CTSD. Furthermore, besides CTSD, PepA can also inhibit the aspartyl protease CTSE, renin and pepsin, arguing the specificity for CTSD. Renin- and pepsin are proteases found in the kidney (renin) and digestive system (pepsin). As the reductions in inflammation were also observed *in vitro*, it is unlikely that either of them are involved in the observed effects in this study. Additionally, CTSE activity was unaffected in plasma, liver and BMDMs after PepA treatment, suggesting that the observed effects are mainly related to the inhibition of CTSD. Yet, it cannot be completely excluded that PepA also influenced other processes that contributed to the observed inflammatory effects in this study.

Enhancing lysosomal exocytosis in several lysosomal storage diseases strongly reduced substrate accumulation, suggesting a pivotal role for this mechanism in the setting of cellular homeostasis^38^. In the context of lipid storage, exocytosis of lysosomes may be a compensatory mechanism to ‘dump’ undigested lipids into the extracellular space as well as an elegant way to conduct signaling to protect the cell from lipid overload. In line with this concept, elevated plasma CTSD levels in mice on HFC diet were diminished after reduction of liver lipid levels. As such, the excretion of lysosomal enzymes may be a secondary consequence of a survival mechanism to protect the cell from lipid overload. Furthermore, changes in lysosomal pH have been shown to alter processing and secretion of CTSD^39^. Notably, intracellular accumulation of poorly degradable, oxidized lipid-protein cross-links was shown to alter the turnover of CTSD, leading to mistargeting of CTSD into the extracellular space^40^. These data suggest that, by targeting specifically the circulating fraction of CTSD, lipid levels may be adjusted.

In summary, our study elucidates a key role for CTSD in the development of NASH. Our data suggest a novel mechanism by which CTSD affects lipid metabolism. Future studies are necessary to determine the exact underlying mechanisms explaining the observed effect in this study and whether other lysosomal enzymes possess similar effects as CTSD. Taken together, targeting CTSD has the potential to become an auspicious, therapeutic strategy for NASH.

## Methods

### Mice, diet and intervention

Experiments were performed according to the Dutch regulation and approved by the Committee for Animal Welfare of Maastricht University. *Ldlr* ^−/−^ mice on a C57BL/6 background were housed under standard conditions and given free access to food and water. Female 12 week-old *Ldlr* ^−/−^ mice were fed either regular chow or a high-fat, high-cholesterol (HFC) diet (SAFE, Augy, France) for 3 weeks and were divided in three groups (n = 11 for each group). The HFC diet contained 21% milk butter, 0.2% cholesterol, 46% carbohydrates and 17% casein. To examine whether proteolytic inhibition of CTSD would decrease hepatic inflammation, *Ldlr* ^−/−^ mice were injected intraperitoneally with pepstatin A (PepA; 50 µg/g body weight; P5318, Sigma, Zwijndrecht, the Netherlands), a proteolytic inhibitor of aspartyl proteases. Mice were injected with DMSO (8%) or PepA two times every week. An overview of all experimental groups is depicted in Supplementary Fig. S1. Collection of blood and tissue specimens, fluorescence-activated cell sorting (FACS), liver histology, plasma alanine transaminase levels (ALT), RNA isolation, cDNA synthesis and qPCR were determined as described previously^4, 15, 41, 42^.

### Bone marrow-derived macrophages (BMDMs)

BMDMs were isolated from the tibiae and femurs of C57BL/6 mice. Cells were cultured in RPMI-1640 (GIBCO Invitrogen, Brede, the Netherlands) with 10% heat-inactivated fetal calf serum (Bondinco B.V. Alkmaar, the Netherlands), penicillin (100 U/ml), streptomycin (100 µg/ml) and L-glutamine 2 mM (GIBCO Invitrogen, Breda, the Netherlands), supplemented with 20% L929-conditioned medium (LCM) for 8–9 days to generate BMDMs. After attachment, macrophages were seeded at 350,000 cells per well in 24-well plates and incubated for 24 h with oxLDL (25 µg/ml) or medium (control). Afterwards, cells were washed and exposed to PepA (10 µg/ml; dissolved in DMSO) or DMSO (0.06%) for 4 h. Next, cells were washed and stimulated with or without LPS (100 ng/ml) for 4 h. Finally, conditioned medium was used for enzyme-linked immunosorbent assays and cells were lysed for mRNA expression analysis. All *in vitro* data are the result of at least three independent experiments.

### Statistical analysis

Data were statistically analyzed by performing two-tailed unpaired *t* test and Area Under the Curve (AUC) analysis using GraphPad Prism version 6 for Windows. Data were expressed as the mean and standard error of the mean (SEM) and were considered significantly different compared to control-treated mice on chow diet (*p ≤ 0.05; **p < 0.01; ***p < 0.001) and compared to control-treated mice on HFC diet (^#^ *p* ≤ 0.05; ^##^ *p* < 0.01; ^###^ *p* < 0.001). Pearson correlation coefficients (*r*) and respective *p* values were calculated to assess the statistical significance of the correlation.

Additional explanation is provided in the *Supplementary Information*.

## Electronic supplementary material

Supplementary Information

## References

[1] Loomba R, Sanyal AJ (2013). The global NAFLD epidemic. Nat Rev Gastroenterol Hepatol.

[2] Ray K (2013). NAFLD-the next global epidemic. Nat Rev Gastroenterol Hepatol.

[3] Nascimbeni F (2013). From NAFLD in clinical practice to answers from guidelines. J Hepatol.

[4] Bieghs V (2012). Internalization of modified lipids by CD36 and SR-A leads to hepatic inflammation and lysosomal cholesterol storage in Kupffer cells. PLoS One.

[5] Bieghs V (2012). Specific immunization strategies against oxidized low-density lipoprotein: a novel way to reduce nonalcoholic steatohepatitis in mice. Hepatology.

[6] Bordon Y (2011). Immune regulation: lysosomes at the heart of inflammation. Nat Rev Immunol.

[7] Sukhova GK (2003). Deficiency of cathepsin S reduces atherosclerosis in LDL receptor-deficient mice. J Clin Invest.

[8] Benes P, Vetvicka V, Fusek M (2008). Cathepsin D–many functions of one aspartic protease. Crit Rev Oncol Hematol.

[9] Khalkhali-Ellis, Z. & Hendrix, M. J. Two Faces of Cathepsin D: Physiological Guardian Angel and Pathological Demon. *Biology and medicine* **6**, doi:10.4172/0974-8369.1000206 (2014). 10.4172/0974-8369.1000206 PMC4318633 25663755

[10] Cataldo AM, Nixon RA (1990). Enzymatically active lysosomal proteases are associated with amyloid deposits in Alzheimer brain. Proc Natl Acad Sci USA.

[11] Hakala JK (2003). Lysosomal enzymes are released from cultured human macrophages, hydrolyze LDL *in vitro*, and are present extracellularly in human atherosclerotic lesions. Arterioscler Thromb Vasc Biol.

[12] Hausmann M (2004). Cathepsin D is up-regulated in inflammatory bowel disease macrophages. Clinical and experimental immunology.

[13] Poole AR (1976). Secretion and localization of cathepsin D in synovial tissues removed from rheumatoid and traumatized joints. An immunohistochemical study. Arthritis and rheumatism.

[14] Fukuo Y (2014). Abnormality of autophagic function and cathepsin expression in the liver from patients with non-alcoholic fatty liver disease. Hepatology research: the official journal of the Japan Society of Hepatology.

[15] Bieghs V (2013). The cholesterol derivative 27-hydroxycholesterol reduces steatohepatitis in mice. Gastroenterology.

[16] Hendrikx T (2013). Macrophage specific caspase-1/11 deficiency protects against cholesterol crystallization and hepatic inflammation in hyperlipidemic mice. PLoS One.

[17] Walenbergh SM (2016). Plasma cathepsin D correlates with histological classifications of fatty liver disease in adults and responds to intervention. Sci Rep.

[18] Bieghs V (2012). LDL receptor knock-out mice are a physiological model particularly vulnerable to study the onset of inflammation in non-alcoholic fatty liver disease. PLoS One.

[19] Vogel C, Marcotte EM (2012). Insights into the regulation of protein abundance from proteomic and transcriptomic analyses. Nat Rev Genet.

[20] Jaensson E (2008). Small intestinal CD103+ dendritic cells display unique functional properties that are conserved between mice and humans. The Journal of experimental medicine.

[21] Randolph GJ (2008). Emigration of monocyte-derived cells to lymph nodes during resolution of inflammation and its failure in atherosclerosis. Current opinion in lipidology.

[22] Conus S (2008). Caspase-8 is activated by cathepsin D initiating neutrophil apoptosis during the resolution of inflammation. The Journal of experimental medicine.

[23] Fischbeck A (2011). Sphingomyelin induces cathepsin D-mediated apoptosis in intestinal epithelial cells and increases inflammation in DSS colitis. Gut.

[24] Menzel K (2006). Cathepsins B, L and D in inflammatory bowel disease macrophages and potential therapeutic effects of cathepsin inhibition *in vivo*. Clinical and experimental immunology.

[25] Li W, Yuan XM (2004). Increased expression and translocation of lysosomal cathepsins contribute to macrophage apoptosis in atherogenesis. Annals of the New York Academy of Sciences.

[26] Figueiredo JL (2015). Selective cathepsin S inhibition attenuates atherosclerosis in apolipoprotein E-deficient mice with chronic renal disease. Am J Pathol.

[27] Maxfield FR, Tabas I (2005). Role of cholesterol and lipid organization in disease. Nature.

[28] Xu X (2013). Obesity activates a program of lysosomal-dependent lipid metabolism in adipose tissue macrophages independently of classic activation. Cell metabolism.

[29] Murphy J, Summer R, Wilson AA, Kotton DN, Fine A (2008). The prolonged life-span of alveolar macrophages. Am J Respir Cell Mol Biol.

[30] Fucho R (2014). ASMase regulates autophagy and lysosomal membrane permeabilization and its inhibition prevents early stage non-alcoholic steatohepatitis. J Hepatol.

[31] Xiong J, Kielian T (2013). Microglia in juvenile neuronal ceroid lipofuscinosis are primed toward a pro-inflammatory phenotype. Journal of neurochemistry.

[32] Siintola E (2006). Cathepsin D deficiency underlies congenital human neuronal ceroid-lipofuscinosis. Brain: a journal of neurology.

[33] Pullinger CR (2002). Human cholesterol 7alpha-hydroxylase (CYP7A1) deficiency has a hypercholesterolemic phenotype. J Clin Invest.

[34] Wang L (2002). Redundant pathways for negative feedback regulation of bile acid production. Developmental cell.

[35] Watanabe M (2004). Bile acids lower triglyceride levels via a pathway involving FXR, SHP, and SREBP-1c. J Clin Invest.

[36] Jonkers IJ, Mohrschladt MF, Westendorp RG, van der Laarse A, Smelt AH (2002). Severe hypertriglyceridemia with insulin resistance is associated with systemic inflammation: reversal with bezafibrate therapy in a randomized controlled trial. The American journal of medicine.

[37] Guicciardi ME, Malhi H, Mott JL, Gores GJ (2013). Apoptosis and necrosis in the liver. Compr Physiol.

[38] Settembre C (2011). TFEB links autophagy to lysosomal biogenesis. Science.

[39] Rosenfeld MG, Kreibich G, Popov D, Kato K, Sabatini DD (1982). Biosynthesis of lysosomal hydrolases: their synthesis in bound polysomes and the role of co- and post-translational processing in determining their subcellular distribution. The Journal of cell biology.

[40] Hoppe G, O’Neil J, Hoff HF, Sears J (2004). Products of lipid peroxidation induce missorting of the principal lysosomal protease in retinal pigment epithelium. Biochim Biophys Acta.

[41] Bieghs, V. *et al*. Role of scavenger receptor A and CD36 in diet-induced nonalcoholic steatohepatitis in hyperlipidemic mice. *Gastroenterology* **138**, 2477–2486, 2486 e2471–2473, doi:10.1053/j.gastro.2010.02.051 (2010). 10.1053/j.gastro.2010.02.051 PMC3114629 20206177

[42] Wouters K (2008). Dietary cholesterol, rather than liver steatosis, leads to hepatic inflammation in hyperlipidemic mouse models of nonalcoholic steatohepatitis. Hepatology.

