# Supplementary material for: Cathepsin D regulates lipid metabolism in murine steatohepatitis
Source: Sci Rep. 2017 Jun 14;7:3494. doi: 10.1038/s41598-017-03796-5 (PMC5471235; doi:10.1038/s41598-017-03796-5)
Supplement: Supplementary file 1 — Supplementary Information [file 41598_2017_3796_MOESM1_ESM.pdf]

Supplementary Information

Cathepsin D regulates lipid metabolism in murine steatohepatitis

Tom Houben, Yvonne Oligschlaeger, Tim Hendrikx, Albert V Bitorina, Sofie MA Walenbergh, Patrick J van Gorp, Marion JJ Gijbels, Silvia Friedrichs, Jogchum Plat, Frank G Schaap, Dieter Lütjohann, Marten H Hofker, Ronit Shiri-Sverdlov

Supplementary Fig. S1

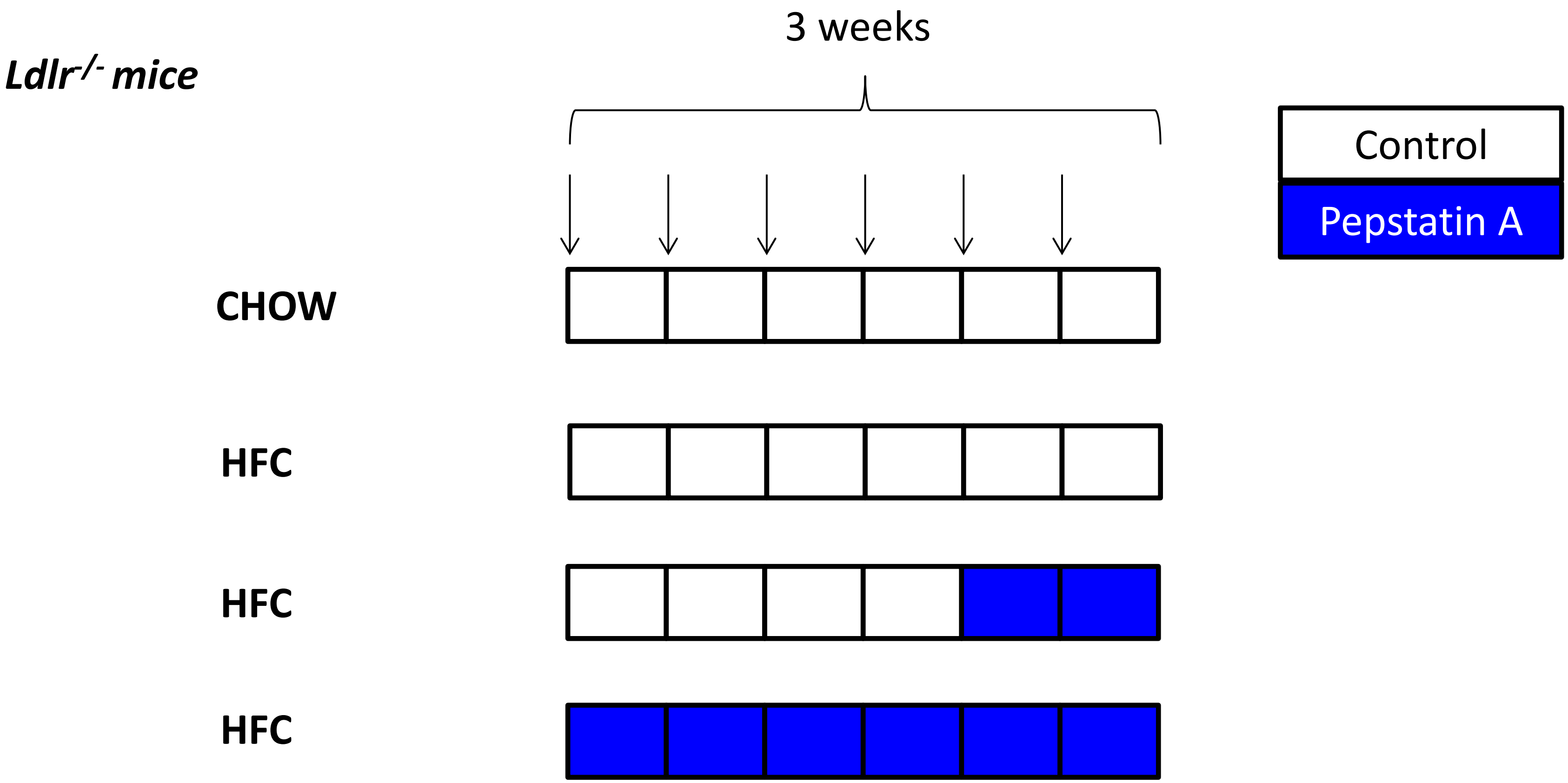

Supplementary Fig. S1: Overview of all experimental groups of the *in vivo* experiment. Arrows indicate time of i.p. injection.

Supplementary Fig. S2

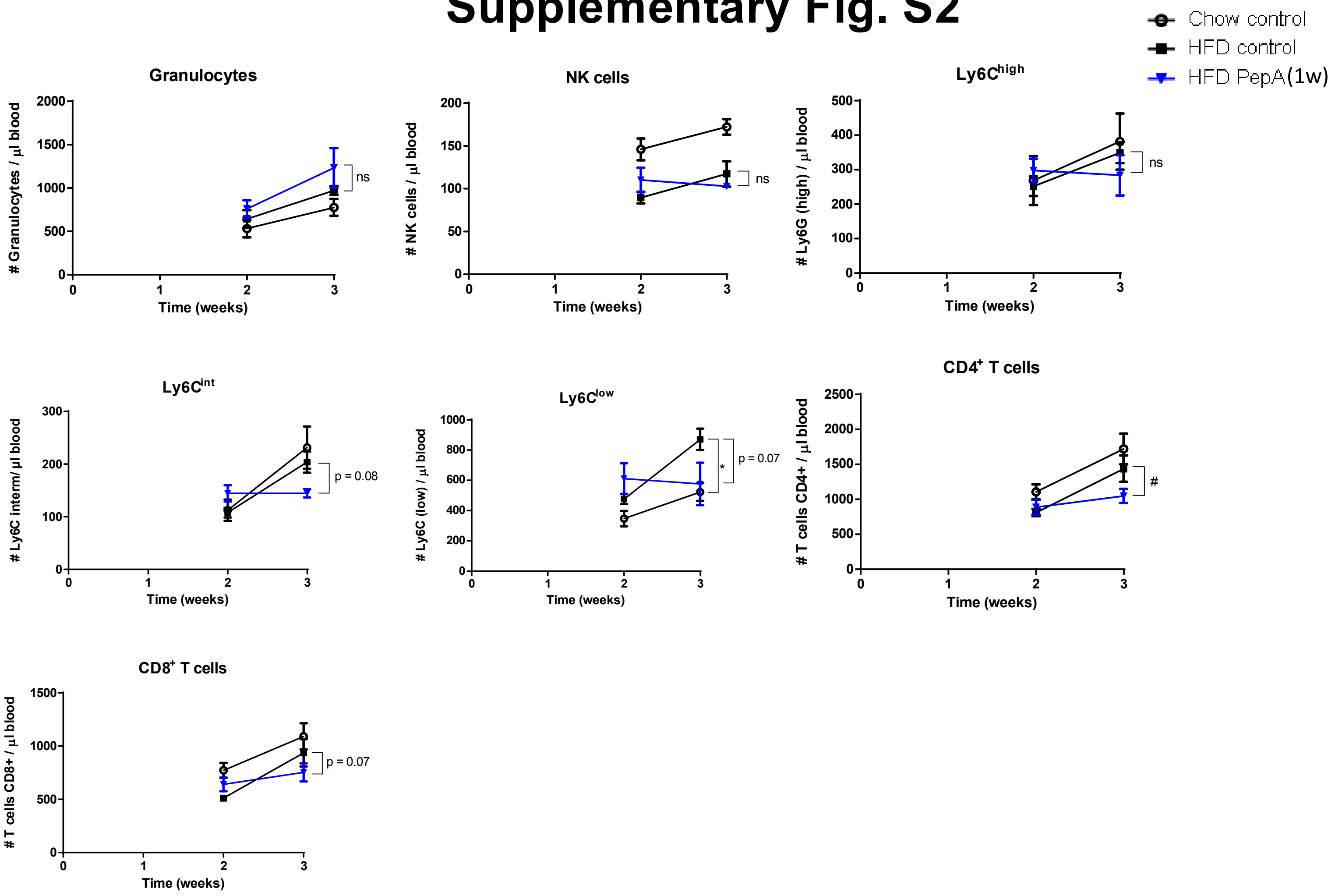

Supplementary Fig. S2: Blood leukocyte profile of granulocytes, NK cells, the monocyte subpopulations (Ly6C^{high}, Ly6C^{int} and Ly6C^{low}) and T cell subpopulations (T-helper (CD4) and cytotoxic T cell (CD8)) by use of two-tailed unpaired *t* test. Error bars represent ± SEM. * Indicates $p < 0.05$ compared to mice on chow diet and $^{\#}$ $p < 0.05$ compared to control-injected mice on HFC diet. n = 4 animals per group. ns, not significant.

Supplementary Fig. S3

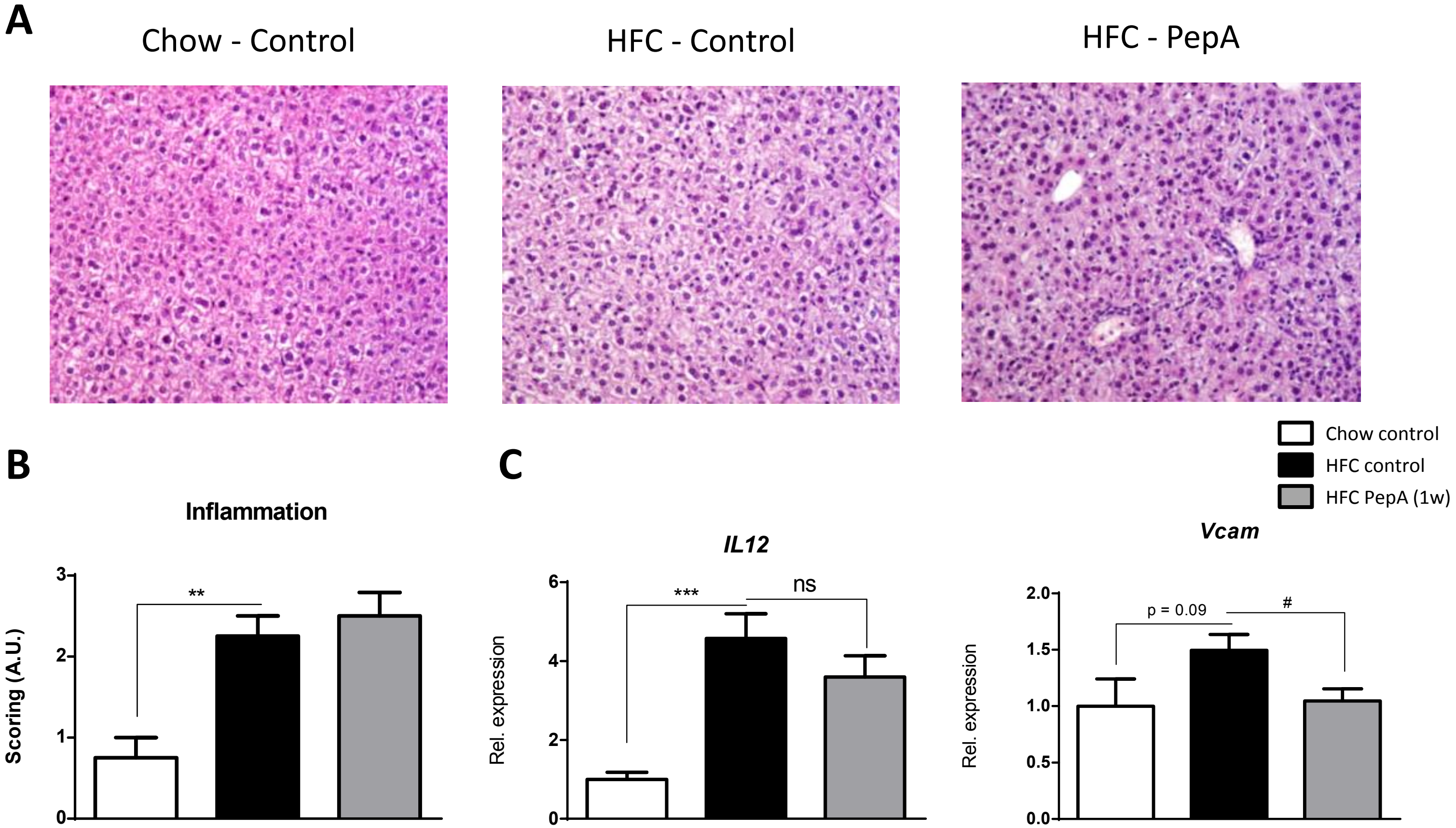

Supplementary Fig. S3: (A) General histology of the liver by a hematoxylin and eosin staining of control- and PepA-injected mice on a chow or HFC diet (original magnification, 200x). (B) Scoring for inflammation of H&E staining. (C) Hepatic gene expression analysis of *IL12* and *Vcam*. Data are shown relative to control mice on chow diet by use of two-tailed unpaired *t*. Error bars represent ± SEM. * Indicates $p = 0.05$, ** $p < 0.01$ and *** $p < 0.001$ compared to mice on chow diet; $^{\#}$ indicates $p < 0.05$ compared to control-injected mice on HFC diet. n = 8-11 animals per group. ns, not significant.

Supplementary Fig. S4

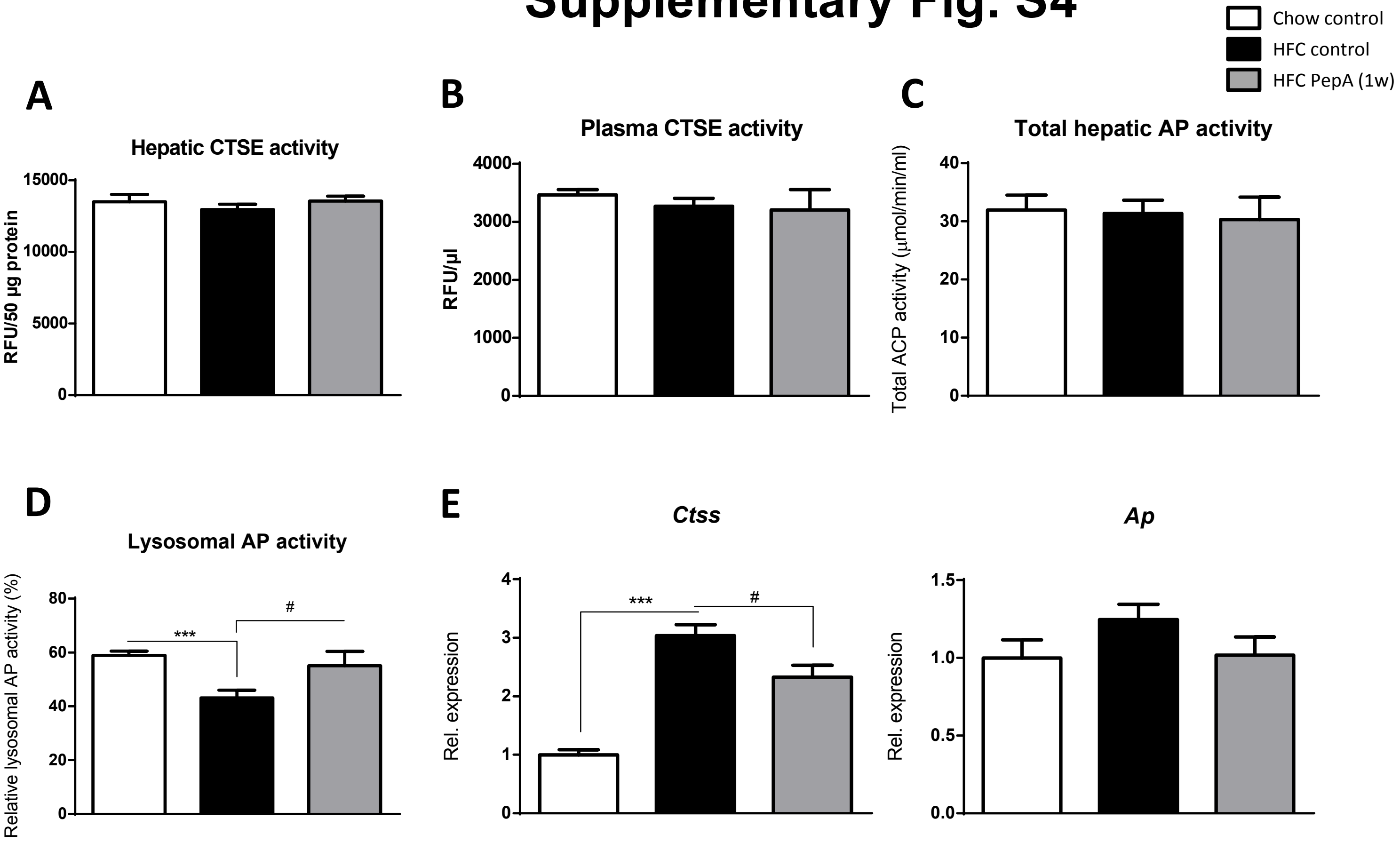

Supplementary Fig. S4: (A, B) Hepatic (A) and plasma (B) activity of CTSE. (C, D)) Total and lysosomal activity of the lysosomal enzyme acid phosphatase (AP) in the liver of control- and PepA-injected mice. (E) Hepatic gene expression analysis of *Ctss* and *Ap*. Data are shown relative to control mice on chow diet by use of two-tailed unpaired *t test.* Error bars represent ± SEM. *** Indicates $p < 0.001$ compared to mice on chow diet; $^{\#}$ indicates $p = 0.05$ compared to control-injected mice on HFC diet by use of two-tailed unpaired t test. n= 9-11 animals in each group.

Supplementary Fig. S5

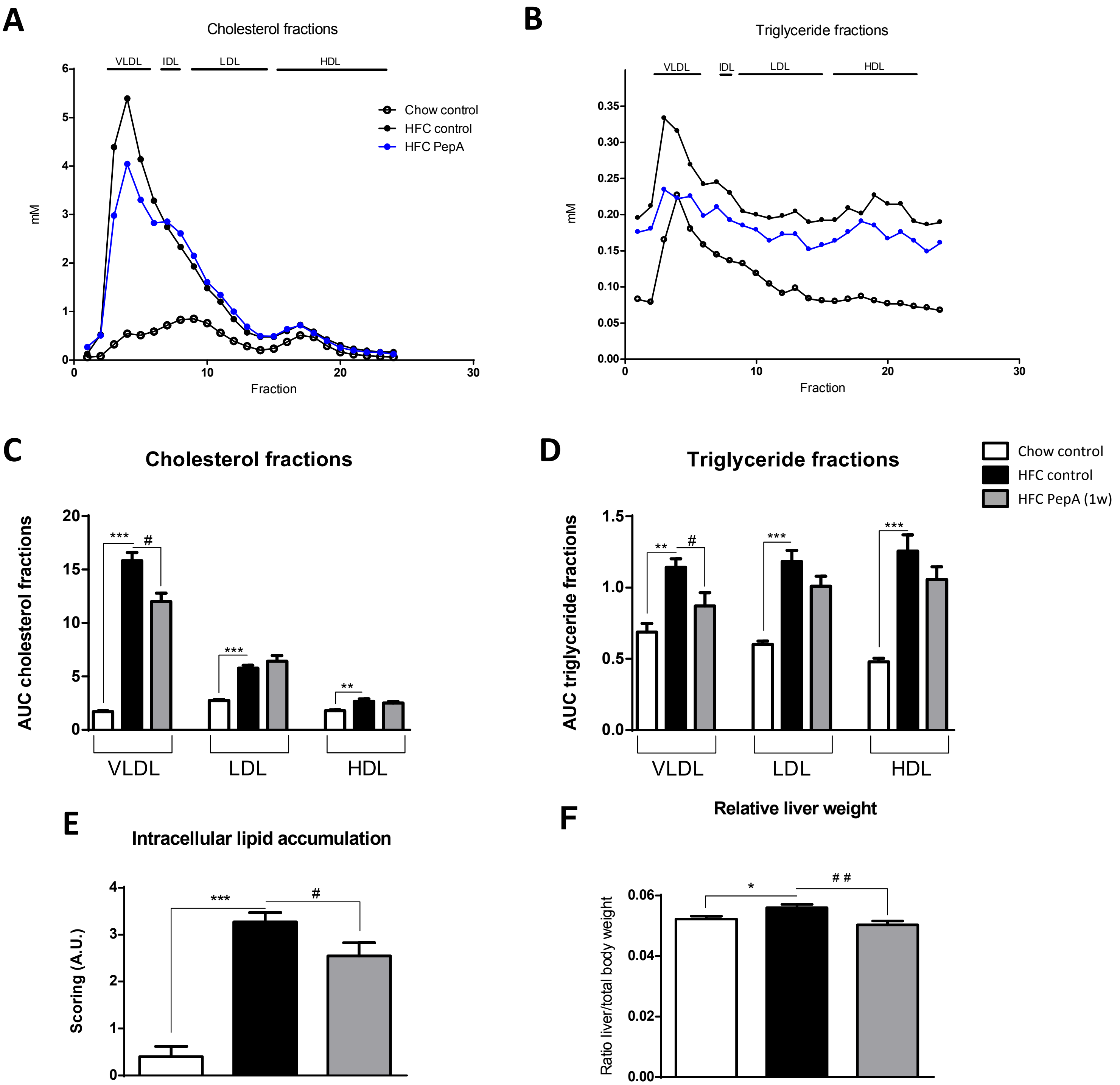

Supplementary Fig. S5: (A, B) Using FPLC, plasma lipid and lipoprotein profiles were analyzed in all experimental groups. Sera of mice were pooled, creating n = 4 for each experimental condition. On the chromatogram, the X-axis represents the fractions present in the mixture as a peak, thereby identifying the different components of the mixture. On the Y-axis, the amount of the different fractions can be read (mM). (C, D) Quantification of plasma cholesterol (C) and triglycerides (D) fractions. (E) Quantification of the Oil Red O staining. (F) Relative liver weight of control- and PepA-injected mice on a chow or HFC diet. * Indicates $p < 0.05$, ** $p < 0.01$ and *** $p < 0.001$ compared to mice on chow diet; $^{\#}$ indicates $p < 0.05$ and $^{\#\#}$ indicates $p < 0.01$ compared to control-injected mice on HFC diet by use of the two-tailed unpaired t test. Error bars represent ± SEM.

Supplementary Fig. S6

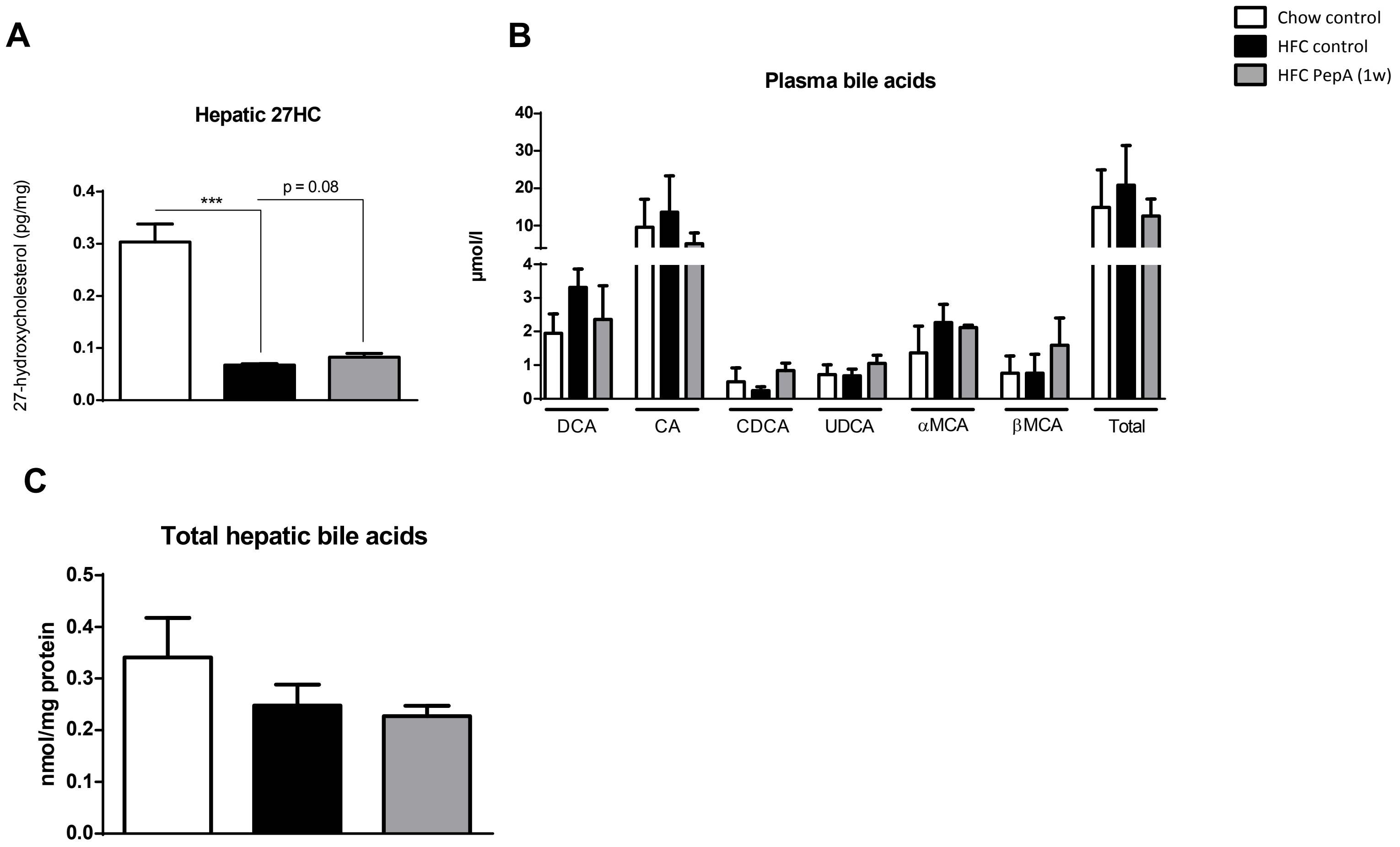

Supplementary Fig. S6: (A) Hepatic concentration of 27-hydroxycholesterol (27HC) by use of two-tailed unpaired *t*. (B) Plasma bile acid levels. (C) Total hepatic bile acid levels. Error bars represent ± SEM. *** Indicates $p < 0.001$ compared to mice on chow diet. Plasma bile acid levels data are pooled from 11 mice, thereby creating n = 4 for each experimental group; n= 9-11 animals in each group. DCA, deoxycholic acid; CA, cholic acid; CDCA, chenodeoxycholic acid; UDCA, ursodeoxycholic acid; aMCA, a-muricholic acid; ßMCA, ß-muricholic acid.

Supplementary Fig. S7

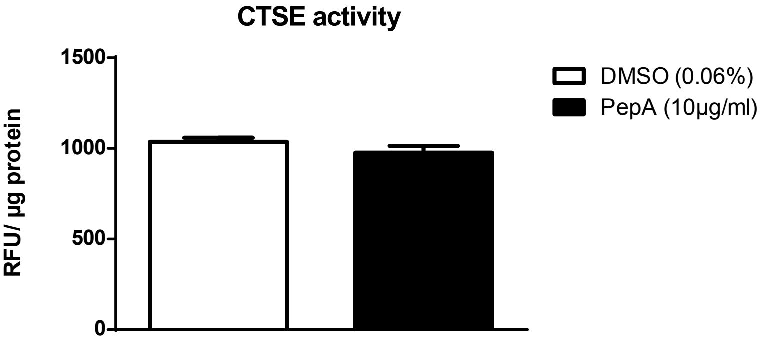

Supplementary Fig. S7: CTSE activity in BMDMs. Error bars represent ± SEM (n = 3)

Supplementary Table S1: Hepatic inflammation after 1 or 3 weeks of pepstatin A treatment

	Chow	HFC		
	**Control**	**Control**	**PepA (1w)**	**PepA (3w)**
**Histology**				
Infiltrating macrophages and neutrophils (Mac1) (# positive cells/mm^2)	32.58 ± 5.191	122.7 ± 11.71***	124.6 ± 13.33	124.8 ± 6.16
T cells (CD3) (# positive cells/mm^2)	71.88 ± 9.59	130.6 ± 22.61*	132.8 ± 13.85	74.77 ± 7.77 **a**
Monocytes/macrophages (CD68) (A.U.)	1.4 ± 0.22	3.5 ± 0.31***	3.00 ± 0.33	3.00 ± 0.26
Macrophage (F4/80) (% positive area)	1.1 ± 0.21	1.3 ± 0.3	1.3 ± 0.2	0.9 ± 0.13
**Gene expression (Rel. Exp.)**				
Tnfα	1.00 ± 0.12	9.52 ± 1.27 ***	5.89 ± 0.91 #	5.25 ± 0.86 #
Caspase1	1.00 ± 0.10	1.98 ± 0.14 ***	1.45 ± 0.14 #	1.36 ± 0.11 ##
Ccl2	1.00 ± 0.14	3.724 ± 0.29 ***	2.00 ± 0.33 ###	1.87 ± 0.47 ##
Cd68	1.00 ± 0.08	3.98 ± 0.22 ***	2.77 ± 0.20 ###	2.52 ± 0.34 ##
Il12	1.00 ± 0.18	4.57 ± 0.63 ***	3.60 ± 0.54	2.55 ± 0.35 #
Vcam	1.00 ± 0.24	1.50 ± 0.14	1.05 ± 0.11 #	1.01 ± 0.15 #
Cd206	1.00 ± 0.09	1.27 ± 0.12	1.03 ± 0.15	0.96 ± 0.12 **b**
Egr2	1.00 ± 0.18	7.06 ± 0.77***	4.48 ± 0.53#	3.87 ± 0.77##
iNos/Arg1	1.00 ± 0.3	7.37 ± 1.1***	6.13 ± 1.23	5.44 ± 0.82
**Protein levels (pg/mg protein)**				
TNFα	38.94 ± 4.19	58.37 ± 11.20	24.62 ± 3.28 ##	18.42 ± 2.27 ##
IL12	0.03 ± 0.01	0.12 ± 0.04 *	0.02 ± 0.01 #	0.02 ± 0.01 #

*Indicates $p < 0.05$ and *** $p < 0.001$ compared to mice on chow diet; # indicates $p \leq 0.05$ and ## $p < 0.01$ compared to control-injected mice on HFC diet; a indicates p = 0.06 and b indicates p = 0.09 compared to control-injected mice on HFC diet by use of two-tailed unpaired *t* test. Standards of error represent ± SEM.

Supplementary Methods

Acid phosphatase activity assay

Hepatic acid phosphatase (AP) activity was determined by the acid phosphatase assay kit (10008051, Cayman Chemical Company, USA). Liver homogenates were diluted 20 times in assay buffer, and transferred to a 96-well plate containing 20 µl assay buffer. Next, 20 µl AP substrate solution was added to each well to initiate the reaction. After 20 minutes incubation at 37°C, the reaction was stopped by adding 100 µl of stop solution to all wells. The absorbance was measured using a Bio-Rad Benchmark 550 Micro-plate reader at 405-414 nm (170-6750XTU, Bio-Rad Laboratories, Veenendaal, the Netherlands).

Cathepsin D activity assay

Cathepsin D activity was measured using the cathepsin D activity assay kit (MBL International, Woburn, MA) according to the manufacturer's protocol. In summary, 50 µg of liver homogenate or 5 µL plasma was lysed in cathepsin D lysis buffer on ice for 10 minutes. Following centrifugation for 5 minutes at top speed, 5 µL of clear cell lysate was transferred to a well of a 96-well plate, and the total volume was made up to 50 µL with cathepsin D cell lysis buffer. To each assay, 52 µL of mastermix (50 µL if CTSD Reaction Buffer and 2 µL of CTSD Substrate) was added, and the plate was incubated at 37ºC for 1 hour. Samples were then measured using a fluorescence plate reader with a 328-nm excitation filter and 460-nm filter. Cathepsin D activity is expressed by the relative fluorescence units.

Cathepsin E activity assay

Cathepsin E activity was measured using the cathepsin E activity assay kit (BioVision, Milpitas, CA) according to the manufacturer's protocol. In summary, 50 µg of liver homogenate, 5 µL plasma or 50 µg bone marrow-derived macrophages was lysed in cathepsin E lysis buffer on ice for 5 minutes. Following centrifugation for 10 minutes at top speed, 5 µL of clear cell lysate was transferred to a well of a 96-well plate, and the total volume was made up to 50 µL with cathepsin E cell lysis buffer. To each assay, 50 µL of Substrate mix (50 µL if CTSE Assay Buffer and 2 µL of CTSE Substrate) was added, and the plate was incubated at 37ºC for 1 hour. Samples were then measured using a fluorescence plate reader with a 320-nm excitation filter and 420-nm filter. Cathepsin E activity is expressed by the relative fluorescence units.

Plasma Lipid Analysis

Both plasma and liver lipid levels were measured with enzymatic color tests (cholesterol CHOD-PAP; 1489232; Roche, Basel, Switzerland; serum triglyceride (TG) determination kit, TR0100; Sigma-Aldrich; NEFAC, ACS-ACOD, 999-75406; Wako Chemicals, Neuss, Germany) according to the manufacturer's protocols on a Benchmark 550 Micro-plate Reader (170-6750XTU; Bio-Rad, Hercules, CA).

Liver Lipid Analysis

Approximately 50 mg of frozen liver tissue was homogenized for 30 seconds at 5000 rpm in a closed tube with 1.0-mm glass beads and 1.0 mL SET buffer (sucrose 250 mmol/L, EDTA 2 mmol/L, and Tris 10 mmol/L). Complete cell destruction was done by 2 freeze-thaw cycles and 3 times passing through a 27-gauge syringe needle and a final freeze-thaw cycle. Protein content was measured with the bicinchoninic acid (BCA) method (23225; Pierce, Rockford, IL). Cholesterol and triglycerides (TG) were measured as described above. Protocols were followed according to the manufacturers' instructions.

Fluorescence-activated cell sorting

FACS analysis was performed as previously described [1]. Graphs shown show the absolute values after 3 weeks (T3) and 2 weeks (T2).

Lipoprotein profiles

Lipoprotein profiles were determined on plasma samples from 4 mice (pooled from 11 to 4 mice of the same experimental group) and determined as described previously [2].

Enzyme-linked immunosorbent assay (ELISA)

TNFα, IL10 and IL12 ELISA assays (resp. 88-7324-88, 88-7105-88 and 88-7121-88, eBioscience, Frankfurt, Germany) were performed on supernatant or liver homogenates according to manufacturer's instructions. Analysis was performed on a Bio-Rad Benchmark 550 Micro-plate reader 450 nm.

Measuring cholesterol precursors, oxysterols and bile acids

Cholesterol precursors, oxysterols and bile acids were measured via highly specific and sensitive gas chromatography-mass spectrometry as described previously [3-5]. Fecal measurement was performed on feces isolated at sacrifice. Total hepatic bile acid levels were specifically determined as described previously [6].

Primer Sequences for Quantitative PCR

Gene	Primer forward	Primer reverse
TNFα	CATCTTCTCAAAATTCGAGTGACAA	TGGGAGTAGACAAGGTACAACCC
CCL2	GCTGGAGAGCTACAAGAGGATCA	TCTCTCTTGAGCTTGGTGACAAAA
Caspase1	GGGACCCTCAAGTTTTGCC	GACGTGTACGAGTGGTTGTATT
CD68	TGACCTGCTCTCTCTAAGGCTACA	TCACGGTTGCAAGAGAAACATG
CD36	GCCAAGCTATTGCGACATGA	AAAAGAATCTCAATGTCCGAGACTTT
NPC2	CGGAGCCCCTGCACTTC	ACAGGGATCGGTGGGACAT
LXRα	CAACAGTGTAACAGGCGCT	TGCAATGGGCCAAGGC
ABCA1	CCCAGAGCAAAAAGCGACTC	GGTCATCATCACTTTGGTCCTTG
PPARγ	TCGCTGATGCACTGCCTATG	GAGAGGTCCACAGAGCTGATT
HmG-CoAR	ACCATGCCATCGATAGAGATAGGA	CGTGCGTTTTCTCCAGGATT
Cyp7a1	CATTACAGAGTGCTGGCCAAGA	CGCAGAGCCTCCTTGATGAT
Cyp27	CTGCACTTCCTGCTGACCAAT	AGGGCCCATGTCAGTGTGTT
IL12	GGAACTACACAAGAACGAGAG	AAGTCCTCATAGATGCTACCA
VCAM	GTGTTGAGCTCTGTGGGTTTTG	TTAATTACTGGATCTTCAGGGAATGAG
CTSD	CCTCCATTCATTGCAAGATACTTG	CACATAGGTGCTGGACTTGTCACT
CTSS	AAAGATTACTGGCTTGTGAAAAACAG	GCAATTCCGCAGTGATTTTTATT
AP	GACCCTAATGGCAACTACCTCTCA	GGGAGCTTGCTTCCCATTG
CD206	TGCAAAGGACTGAAAGGAAACC	CCAGTCCAGGCATTGAAAGTG
EGR2	CTACCCGGTGGAAGACCTC	AATGTTGATCATGCCATCTCC
iNOS	GCAAACCCAAGGTCTACGTTCA	CCTCATTGGCCAGCTGCTT
Arg1	CATGGGCAACCTGTGTCCTT	CGATGTCTTTGGCAGATATGCA

1 Hendrikx, T. *et al.* Bone marrow-specific caspase-1/11 deficiency inhibits atherosclerosis development in Ldlr(-/-) mice. *FEBS J* **282**, 2327-2338, doi:10.1111/febs.13279 (2015).

2 Plat, J. *et al.* Protective role of plant sterol and stanol esters in liver inflammation: insights from mice and humans. *PLoS One* **9**, e110758, doi:10.1371/journal.pone.0110758 (2014).

3 Lutjohann, D. *et al.* Profile of cholesterol-related sterols in aged amyloid precursor protein transgenic mouse brain. *J Lipid Res* **43**, 1078-1085 (2002).

4 Schierwagen, R. *et al.* Seven weeks of Western diet in apolipoprotein-E-deficient mice induce metabolic syndrome and non-alcoholic steatohepatitis with liver fibrosis. *Sci Rep* **5**, 12931, doi:10.1038/srep12931 (2015).

5 Czubayko, F., Beumers, B., Lammsfuss, S., Lutjohann, D. & von Bergmann, K. A simplified micro-method for quantification of fecal excretion of neutral and acidic sterols for outpatient studies in humans. *J Lipid Res* **32**, 1861-1867 (1991).

6 Modica, S., Murzilli, S. & Moschetta, A. Characterizing Bile Acid and Lipid Metabolism in the Liver and Gastrointestinal Tract of Mice. *Curr Protoc Mouse Biol* **1**, 289-321, doi:10.1002/9780470942390.mo100226 (2011).
